# Lower BCL11B expression is associated with adverse clinical outcome for patients with myelodysplastic syndrome

**DOI:** 10.1186/s40364-021-00302-y

**Published:** 2021-06-10

**Authors:** Xin Huang, Cunte Chen, Mengjun Zhong, Suxia Geng, Yujie Zhao, Minming Li, Chenxin Deng, Lingji Zeng, Ping Wu, Zesheng Lu, Jianyu Weng, Xin Du, Yangqiu Li

**Affiliations:** 1 Department of Hematology, Guangdong Provincial People’s Hospital, Guangdong Academy of Medical Sciences, 510080 Guangzhou, PR China; 2 Key Laboratory for Regenerative Medicine of Ministry of Education, Institute of Hematology, Jinan University, 510632 Guangzhou, PR China

**Keywords:** BCL11B, prognosis, myelodysplastic syndrome, immune cell

## Abstract

Myelodysplastic syndrome (MDS) is an aggressive and genetically heterogeneous disease with poor prognosis. Cellular immune disorder is a common characteristic of this disease and is thought to be related to clinical outcome. Alterations in T cell clonal expansion and T cell dysfunction has been detected in MDS patients. Little is known about whether there are immune biomarkers to evaluate the T cell alterations with clinical outcome. Previous studies have demonstrated that B-cell leukemia/lymphoma 11B (BCL11B) plays an important role in regulating T cell development and proliferation. In this study, the prognostic value of BCL11B for MDS patients was explored by analyzing RNA-seq data from 270 patients in two datasets in the Gene Expression Omnibus (GEO) database and real-time quantitative PCR data (qRT-PCR) of 31 bone marrow (BM) samples of MDS and 6 BM samples of patients with MDS progress to secondary acute myeloid leukemia (sAML) from our clinical center. The results demonstrated that BCL11B is significantly down-regulated in MDS patients as compared with healthy individuals (HIs). Importantly, lower BCL11B expression was found in MDS patients who were of high/very high risk, older than 60 y, or male and patients with sAML. Furthermore, low BCL11B expression appeared to be associated with poor overall survival (OS) for MDS patients, though the data were not yet significant enough at this point. In addition, BCL11B low-expressing MDS patients had shorter restricted mean survival time (RMST) than those with high BCL11B expression. Interestingly, BCL11B positively correlated with naive and activated memory CD4 + T cells, CD8 + T cells, and the T cell receptor complex genes CD3E and CD3G, but it negatively correlated with regulatory T cells (Treg). Additionally, co-occurrence of low BCL11B expression and CD3E and CD3G was associated with poor OS and shorter RMST. In conclusion, lower BCL11B expression in BM samples of MDS patients was associated with adverse clinical outcome.

**Supplementary Information:**

The online version contains supplementary material available at 10.1186/s40364-021-00302-y.

To the Editor,

Myelodysplastic syndrome (MDS) is an aggressive hematological disorder that displays hematologic and prognostic heterogeneity, illustrating the need for accurate mechanisms, prognostic biomarkers, and individualized therapies [1–3]. MDS progression appears to be associated with changes in the immune microenvironment that inhibit effective anti-tumor responses [4]. Anti-tumor effector T cells could be identified in the peripheral blood and BM of MDS patients, and this is considered to be favorable for clinical outcome; however, their mechanisms involved in promoting anti-tumor immunity have not been fully investigated [5–7]. B-cell leukemia/lymphoma 11B (BCL11B) plays an important role in regulating the development and maintenance of T cell activation [8]. Lower expression of BCL11B results in T cell dysfunction and is a reason for T cell deficiency in leukemia [9]. However, little is known about the impact of BCL11B expression on the prognosis of MDS patients. In this study, two large datasets containing transcriptome sequencing data from 270 MDS patients and 73 healthy individuals (HIs) from the Gene Expression Omnibus (GEO) database [10], and 31 bone marrow (BM) samples of MDS and 6 BM samples of patients with MDS progress to secondary acute myeloid leukemia (sAML) from our clinical center (Table S1) were used to explore the prognostic value of BCL11B and T cell activity for MDS patients.

In this study, the gene expression levels of BM samples from our clinical center were confirmed by quantitative real-time PCR, and the primers were listed in Table S2. We first found that BCL11B was significantly down-regulated in MDS patients compared with HIs in the GSE13159 dataset (*P* < 0.001; Fig. 1 A). It is known that males, elderly age, and high-risk MDS patients have poor prognoses [1, 11]. Notably, MDS patients with high/very high risk (*n* = 16) and patients with sAML (*n* = 6) had lower BCL11B expression than those with low- (*n* = 8) and intermediate-risk (*n* = 7) MDS patients in the BM samples (*P* = 0.027; Fig. 1B). Moreover, compared with patients who were younger than 60 y, those older than 60 y had lower BCL11B expression (*P* = 0.048; Fig. 1B). In addition, down-regulation of BCL11B was found in male patients as compared with female patients (*P* = 0.027; Fig. 1B). Importantly, low BCL11B expression appeared to be correlated with poor overall survival (OS) for MDS patients in the GSE114922 dataset (5-year OS rate: 27 % vs. 71 %, *P* = 0.094; Fig. 1 C and S1A), though the data were not yet significant enough at this point. Furthermore, patients with low BCL11B expression had shorter restricted mean survival time (RMST) than those with high BCL11B expression (5-year RMST: 1,189 vs. 1,437 days; Fig. 1 C). These results indicate that down-regulation of BCL11B may play an important role in the progression of MDS.

**Fig. 1:**
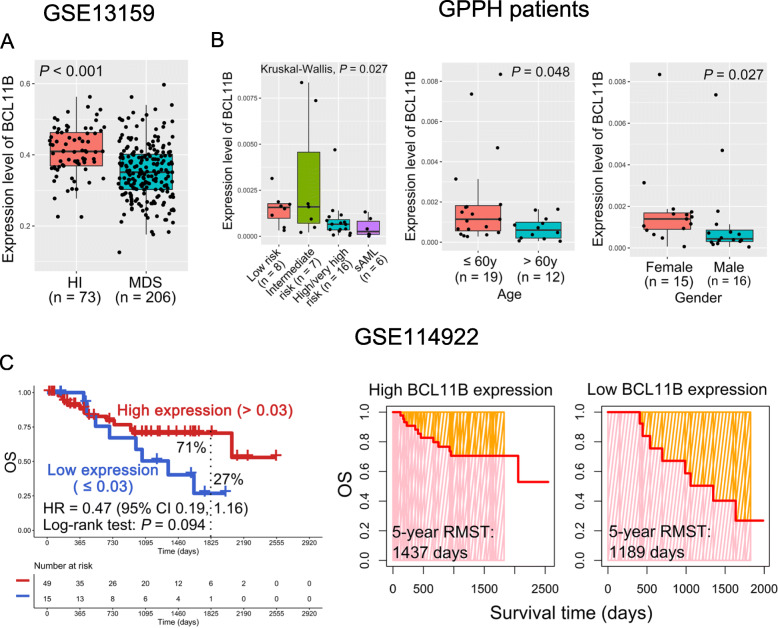
BCL11B down-regulation is associated with poor prognosis in MDS patients. **A** BCL11B expression level in healthy individuals (HIs) and patients with MDS in the GSE13159 dataset. **B** Differences in BCL11B expression among different risk stratifications (left panel), age (middle panel), and gender (right panel) in bone marrow (BM) samples from our clinical center. **C** The overall survival (OS) (left panel) and restricted mean survival time (RMST) (right panel) for the high and low BCL11B expression groups in the GSE114922 dataset. GDPH, Guangdong Provincial People’s Hospital; sAML, secondary acute myeloid leukemia

To elucidate differences in BCL11B expression in different T cell subsets that primarily serve a regulatory role, we analyzed the correlation between BCL11B and T cell subsets. The results demonstrated that BCL11B mainly had a positive correlation with naive and activated memory CD4 + and CD8 + T cells (R > 0, *P* < 0.001), and it had a negative correlation with Tregs in the GSE13159 dataset (*R* = -0.17, *P* = 0.017; Fig. 2 A). Interestingly, BCL11B-related genes were enriched in the T cell receptor signaling pathway in the GSE13159 dataset (*P* = 0.047, Fig. S2). Moreover, BCL11B was positively correlated with the T cell receptor (TCR) complex genes CD3D, CD3E, and CD3G in both the GSE13159 and GSE114922 datasets (R > 0, *P* < 0.01; Fig. 2B). Notably, high expression of CD3E and CD3G appeared to be associated with favorable OS for MDS patients in the GSE114922 dataset (*P* < 0.1; Fig. 2 C and S1B-D), though the data were not yet significant enough at this point. Moreover, the expression levels of BCL11B and CD3E or CD3G had a positive correlation (R > 0, *P* < 0.05; Fig. 2D). We then further analyzed the contribution of the co-expression patterns of BCL11B, CD3E, and CD3G for the OS of MDS [12, 13]. Significantly, MDS patients who were BCL11B^low^CD3E^low^CD3G^low^, BCL11B^low^, CD3E^low^, or CD3G^low^ had a worse OS rate than those who were BCL11B^high^CD3E^high^CD3G^high^ (4-year OS rate: 25 % vs. 57 % vs. 84 %, *P* = 0.040), and they also had a shorter RMST (4-year RMST: 1,072 vs. 1,086 vs. 1,283 days; Fig. 2E).

**Fig. 2:**
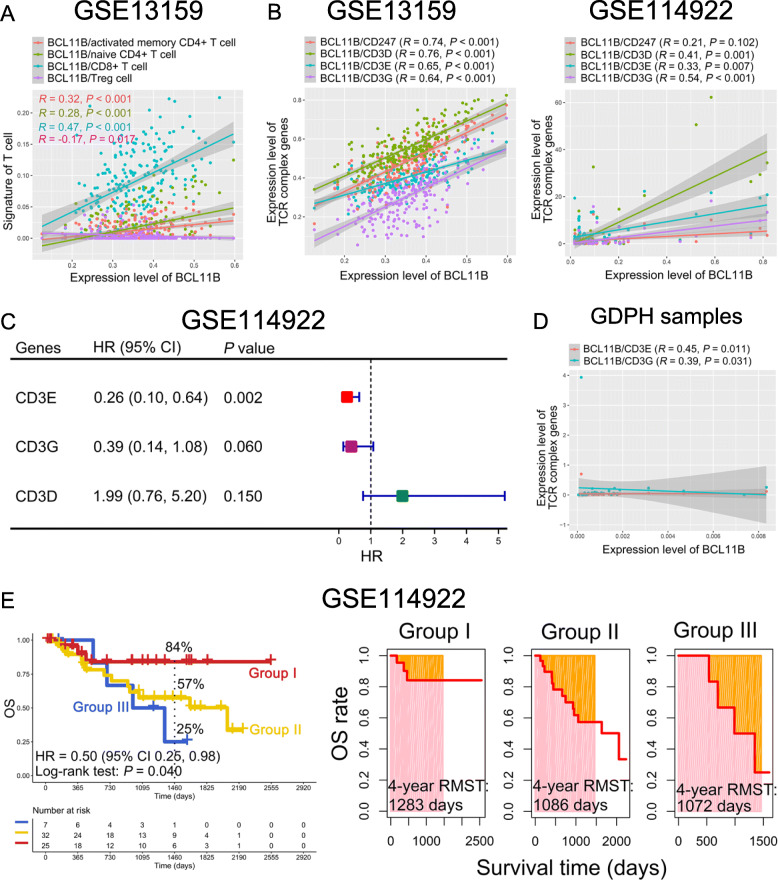
Relationship between BCL11B and immune infiltrating lymphocytes and CD3 complex genes. **A** T cell subsets were correlated with BCL11B in the GSE13159 dataset. **B** Correlation between BCL11B and CD3 complex genes in the GSE13159 (left panel) and GSE114922 (right panel) datasets. **C** Impact of the CD3D, CD3E, and CD3G expression levels on the OS of MDS patients in the GSE114922 dataset. **D** Correlation between BCL11B and CD3E or CD3G in bone marrow (BM) samples. **E** Impact of the combination of BCL11B, CD3E, and CD3G on OS and RMST in MDS patients. Group I: BCL11B^high^CD3E^high^CD3G^high^; Group II: BCL11B^low^, CD3E^low^, or CD3G^low^; Group III: BCL11B^low^CD3E^low^CD3G^low^. GDPH, Guangdong Provincial People’s Hospital

In conclusion, lower BCL11B expression in BM samples of MDS patients was associated with adverse clinical outcome.

## Supplementary information

Additional file 1: **Fig. S1.** The optimal cut-points of BCL11B (**A**), CD3G (**B**), CD3E (**C**) and CD3D (**D**) were obtained.

Additional file 2: **Fig. S2.** BCL11B-related genes were enriched in the Kyoto Encyclopedia of Genes and Genomes (KEGG) pathways of cancer. Based on the two groups with low and high expression of BCL11B, using the “limma” package for differential gene analysis, 4038 genes with *P*-value < 0.05 were identified. Then, “DOSE”, “org.Hs.eg.db”, “topGO” and “clusterProfiler” packages were used to obtain the cancer-related KEGG pathways enriched by BCL11B related genes.

Additional file 3: **Table S1. **Clinical information of the MDS patients.

Additional file 4: **Table S2.** Primers for qRT-PCR.

Additional file 5: Materials and Methods.

## Data Availability

Data available upon request.

## Abbreviations

BCL11B: B-cell chronic lymphocytic leukemia/lymphoma 11B
BM: bone marrow
GEO: Gene Expression Omnibus
GDPH: Guangdong Provincial People’s Hospital
HI: healthy individuals
MDS: myelodysplastic syndrome
OS: overall survival
RMST: restricted mean survival time
sAML: secondary acute myeloid leukemia
