# Supplementary figures and images for: Lower BCL11B expression is associated with adverse clinical outcome for patients with myelodysplastic syndrome
Source: Biomark Res. 2021 Jun 10;9:46. doi: 10.1186/s40364-021-00302-y (PMC8193904; doi:10.1186/s40364-021-00302-y)

A

BCL11B

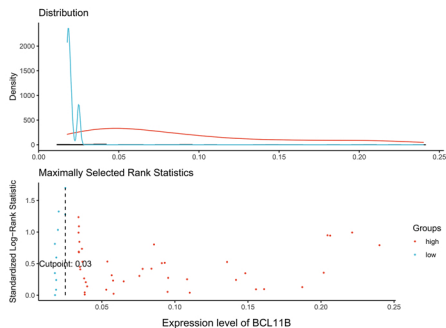

B

CD3G

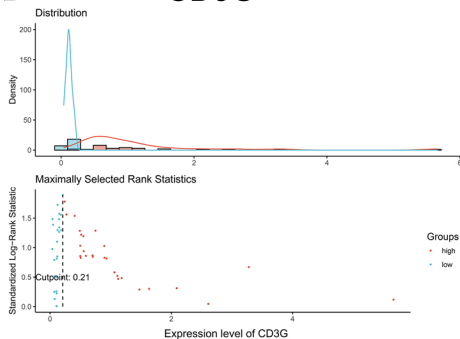

C

CD3E

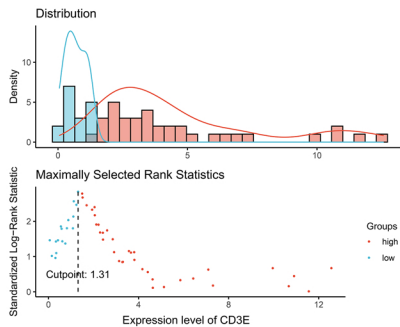

D

CD3D

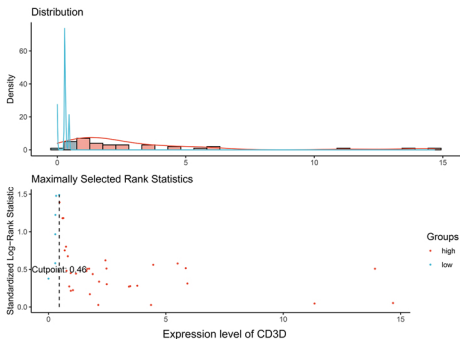

Supplement: Supplementary file 1 — Fig. S1. The optimal cut-points of BCL11B (A), CD3G (B), CD3E (C) and CD3D (D) were obtained. [file 40364_2021_302_MOESM1_ESM.pdf]

# GSE114922

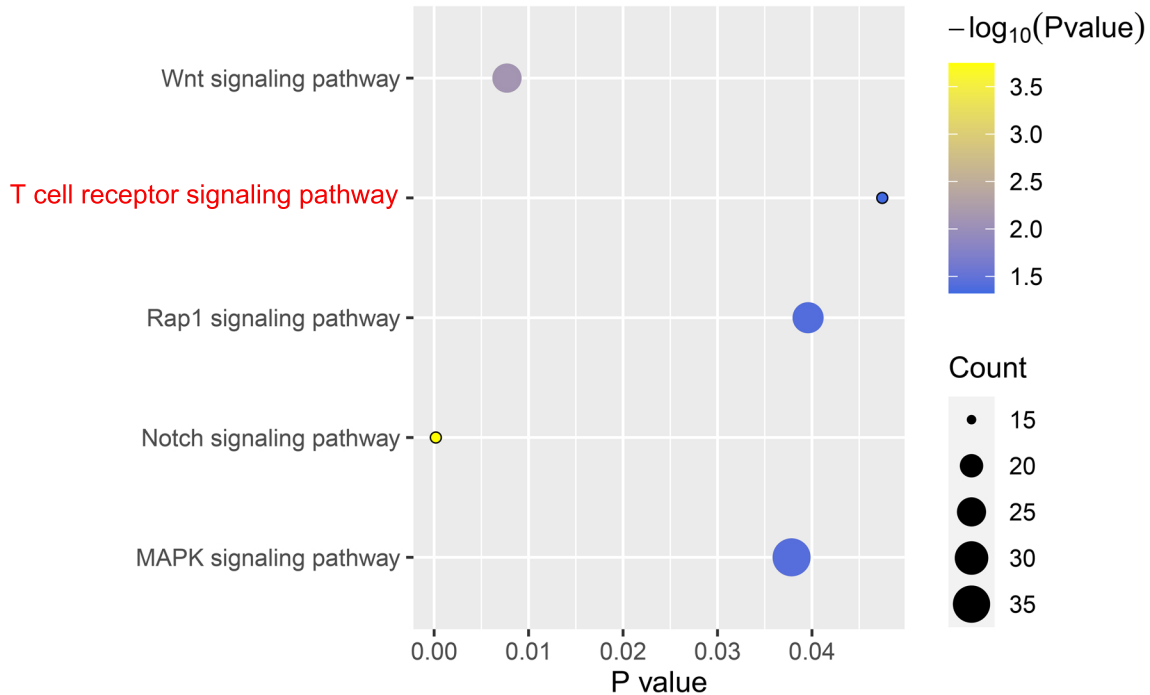

Supplement: Supplementary file 2 — Fig. S2. BCL11B-related genes were enriched in the Kyoto Encyclopedia of Genes and Genomes (KEGG) pathways of cancer. Based on the two groups with low and high expression of BCL11B, using the “limma” package for differential gene analysis, 4038 genes with P-value < 0.05 were identified. Then, “DOSE”, “org.Hs.eg.db”, “topGO” and “clusterProfiler” packages were used to obtain the cancer-related KEGG pathways enriched by BCL11B related genes. [file 40364_2021_302_MOESM2_ESM.pdf]
