# Supplementary material for: Lower BCL11B expression is associated with adverse clinical outcome for patients with myelodysplastic syndrome
Source: Biomark Res. 2021 Jun 10;9:46. doi: 10.1186/s40364-021-00302-y (PMC8193904; doi:10.1186/s40364-021-00302-y)
Supplement: Supplementary file 3 — Table S1. Clinical information of the MDS patients. [file 40364_2021_302_MOESM3_ESM.docx]

**Table S1.** Clinical information of the MDS patients.

| **Variables** | **GSE13159** | **GSE114922** | **BM samples** |
| --- | --- | --- | --- |
| Number | 206 | 64 | 37 |
| Sample source | BM | BM | BM |
| Cancer type, n (%) |  |  |  |
| MDS | 206 (100) | 64 (100) | 31 (83.8) |
| sAML | 0 (0) | 0 (0) | 6 (16.2) |
| Gender, n (%) |  |  |  |
| Female | NA | 26 (40.6) | 15 (48.4) |
| Male | NA | 38 (59.4) | 16 (51.6) |
| Age, years, median (range) | NA | 67 (26, 85) | 58 (23, 81) |
| Risk stratification by IPSS-R, n (%) |  |  |  |
| Low/very low | NA | 29 (45.3) | 8 (25.8) |
| Intermediate | NA | 15 (23.4) | 7 (22.6) |
| High/very high | NA | 16 (25.0) | 16 (51.6) |
| Unknown | NA | 4 (6.3) | 0 (0) |
| OS, days, median  (range) | NA | 942 (22, 2549) | NA |
| Status (alive / death), n | NA | 44 / 20 | NA |

MDS, myelodysplastic syndrome; NA, not available; IPSS-R, revised international prognostic scoring system; OS, overall survival; sAML, secondary acute myeloid leukemia.
