# Supplementary material for: Lower BCL11B expression is associated with adverse clinical outcome for patients with myelodysplastic syndrome
Source: Biomark Res. 2021 Jun 10;9:46. doi: 10.1186/s40364-021-00302-y (PMC8193904; doi:10.1186/s40364-021-00302-y)
Supplement: Supplementary file 4 — Table S2. Primers for qRT-PCR. [file 40364_2021_302_MOESM4_ESM.docx]

**Table S2.** Primers for qRT-PCR.

| **Targets** | **Sequence 5' - 3'** |
| --- | --- |
| GAPDH-F | AAGGTCGGAGTCAACGGATT |
| GAPDH-R | CTGGAAGATGGTGATGGGATT |
| BCL11B-F | ATGTCCCGCCGCAAACAGG |
| BCL11B-R | GGCTCGGACACTTTCCTGAGC |
| CD3G-F | GGGATGTATCAGTGTAAAGG |
| CD3G-R | CAGCAATGAAGTAGACCC |
| CD3E-F | TCCCAACCCAGACTATGAGC |
| CD3E-R | CAAGACTAGCCCAGGAAACAG |
