# Supplementary material for: Lower BCL11B expression is associated with adverse clinical outcome for patients with myelodysplastic syndrome
Source: Biomark Res. 2021 Jun 10;9:46. doi: 10.1186/s40364-021-00302-y (PMC8193904; doi:10.1186/s40364-021-00302-y)
Supplement: Supplementary file 5 — Materials and Methods. [file 40364_2021_302_MOESM5_ESM.doc]

**Materials and Methods**

**Gene Expression Omnibus database (GEO) datasets**

RNA sequencing data of 206 MDS patient and 73 healthy individual (HI) BM samples in the GSE13159 dataset [1, 2] and 64 MDS patients with complete prognostic information and survival time greater than 0 in the GSE114922 dataset [3] were obtained from the GEO database (<https://www.ncbi.nlm.nih.gov/geo/>). The expression of repeated genes was expressed as mean values. Clinical information including sample source, gender, age, risk stratification by revised international prognostic scoring system (IPSS-R), survival time, and events are listed in **Table S1**. The GEO database is publicly available; thus, approval from the local ethics committee was not required.

**BM samples**

A total of 31 BM samples from de novo MDS patients and 6 BM samples from patients with MDS progress to secondary acute myeloid leukemia (sAML) were obtained at the Guangdong Provincial People's Hospital. Corresponding clinical characteristics were also collected, including gender, age, and risk stratification by IPSS-R (**Table S1)**. This study was approved by the Ethics Committee of Guangdong Provincial People's Hospital. All participants provided written informed consent.

### Isolation of RNA and quantitative real-time PCR (qRT-PCR)

Isolation of total RNA and reverse transcription of RNA into cDNA were performed according to the manufacturer's protocol [4]. Then, the expression levels of BCL11B, CD3E, and CD3G were quantified according to the manufacturer's protocol for the qRT-PCR kit (TIANGEN, China), and GAPDH was used as an internal control using the Real-Time System (Bio-Rad, USA) [5, 6]. The primers used for qRT-PCR are listed in **Table S2**. The expression levels of BCL11B, CD3E, and CD3G are presented as 2^-ΔCT^ values.

**CIBERSORT**

CIBERSORT (<https://cibersort.stanford.edu/>) [7] was used to analyze the relative expression levels of 547 genes in each sample based on their gene expression profile, which could predict the proportion of 22 types of tumor-infiltrating immune cells (TIICs) in each sample, namely naive B cells, memory B cells, plasma cells, CD8+ T cells, naive CD4+ T cells, CD4+ resting memory T cells, CD4+ memory-activated T cells, follicular helper T cells, Treg cells, γδ T cells, resting natural killer cells, activated natural killer cells, monocytes, M0 macrophages, M1 macrophages, M2 macrophages, resting dendritic cells, activated dendritic cells, resting mast cells, activated mast cells, eosinophils, and neutrophils. The expression signature of all genes in the GSE13159 dataset were included in the CIBERSORT analysis to obtain the proportion of 22 TIICs.

**Statistical analysis**

All statistical analyses were performed by R software (version 4.0.2, <https://www.r-project.org/>). Differences between two groups of quantitative data were determined by the Mann-Whitney-Wilcoxon test, and differences among three groups of quantitative were compared by the Kruskal-Wallis test. The "surv_cutpoint" function in the "survminer" package was used to determine the optimal prognostic cut-off value of a gene (**Fig. S1**). Kaplan-Meier curves were compared by the log-rank test. Correlations between two groups of data were represented with Spearman’s coefficient. The "survRM2" package was used to obtain the restricted mean survival time (RMST). A two-tailed *P* value < 0.05 was considered statistically significant.

**References**

1. Haferlach T, Kohlmann A, Wieczorek L, Basso G, Kronnie GT, Béné MC, De Vos J, Hernández JM, Hofmann WK, Mills KI, et al. Clinical utility of microarray-based gene expression profiling in the diagnosis and subclassification of leukemia: report from the International Microarray Innovations in Leukemia Study Group. J Clin Oncol. 2010; 28(15):2529-37.

2. Kohlmann A, Kipps TJ, Rassenti LZ, Downing JR, Shurtleff SA, Mills KI, Gilkes AF, Hofmann WK, Basso G, Dell'orto MC, et al. An international standardization programme towards the application of gene expression profiling in routine leukaemia diagnostics: the Microarray Innovations in LEukemia study prephase. Br J Haematol. 2008; 142(5):802-7.

3. Pellagatti A, Armstrong RN, Steeples V, Sharma E, Repapi E, Singh S, Sanchi A, Radujkovic A, Horn P, Dolatshad H, et al. Impact of spliceosome mutations on RNA splicing in myelodysplasia: dysregulated genes/pathways and clinical associations. Blood. 2018; 132(12):1225-40.

4. Zeng C, Liu S, Lu S, Yu X, Lai J, Wu Y, Chen S, Wang L, Yu Z, Luo G, et al. The c-Myc-regulated lncRNA NEAT1 and paraspeckles modulate imatinib-induced apoptosis in CML cells. Mol Cancer. 2018; 17:130.

5. Chen C, Xu L, Gao R, Wang S, Zhang Y, Wang C, Zeng C, Y L. Transcriptome-Based CoExpression of BRD4 and PD-1/PD-L1 Predicts Poor Overall Survival in Patients With Acute Myeloid Leukemia. Front Pharmacol. 2021; 11:582955.

6. Chen C, Liang C, Wang S, Chio CL, Zhang Y, Zeng C, Chen S, Wang C, Li Y. Expression patterns of immune checkpoints in acute myeloid leukemia. J Hematol Oncol. 2020; 13(1):28.

7. Newman AM, Liu CL, Green MR, Gentles AJ, Feng W, Xu Y, Hoang CD, Diehn M, Alizadeh AA. Robust enumeration of cell subsets from tissue expression profiles. Nat Methods. 2015; 12(5):453-7.
